# Cord Blood Karyotyping: A Safe and Non-Invasive Method for Postnatal Testing of Assisted Reproductive Technology Children

**DOI:** 10.22074/ijfs.2016.5046

**Published:** 2016-09-05

**Authors:** Shabnam Zarei Moradi, Najmehsadat Masoudi, Anahita Mohseni Meybodi, Khadijeh Anisi Hemaseh, Ramin Mozafari Kermani, Abolhasan Shahzadeh Fazeli, Hamid Gourabi

**Affiliations:** 1 Department of Genetics , Reproductive Biomedicine Research Center, Royan Institute for Reproductive Biomedicine, ACECR, Tehran, Iran; 2 Child Health and Development Research Center, Iran Medical Science Branch of ACECR, Tehran, Iran

**Keywords:** Assisted Reproductive Technics, Chromosomal Abnormality, Umbilical Cord, Blood, karyotype

## Abstract

**Background:**

To verify the hypothesis that the incidence of chromosomal abnormalities increases in babies conceived by different assisted reproduction procedures. The availability of the umbilical cord blood encouraged us to study this hypothesis via this method.

**Materials and Methods:**

This is a descriptive study, umbilical cord blood samples of assisted reproductive technology (ART) children were analyzed with standard cytogenetic techniques (G banding). Karyotyping was possible in 109 cases.

**Results:**

The number of abnormal cases was four (3.7%), among which, three cases (2.8%) were inherited and only 1 case (0.9%) was a de novo translocation. In total, the incidence of de novo chromosomal abnormalities was in the range observed in all live births in the general population (0.7-1%).

**Conclusion:**

No significant difference in the incidence of chromosomal abnormality was found between ART and naturally conceived babies. To date, several studies have examined the medical and developmental outcome of ART children and still have not reached a definite conclusion. Genetic counseling is recommended as an integral part of planning of treatment strategies for couples wishing to undergo ART.

## Introduction

Assisted reproduction technology (ART) has provided great benefit for millions of couples who struggle with infertility. The definition of ART varies widely, but the US Center for Disease Control and Prevention (CDC) defines it as all fertility treatment in which both eggs and sperms are handled (1). Accordingly, in this study, we defined intrauterine insemination (IUI) babies along with *in vitro* fertilization (IVF) and intra-cytoplasmic sperm injection (ICSI) babies as one group. The growing use of ART has dramatically increased the possibility of conceiving babies from infertile couples. Since then, the safety of these methods alongside the associated long-term impacts on the health of children has been a major concern. There are evidences of greater risks of low birth weight, preterm delivery (2, 3), cerebral palsy (4), and major birth defects (5) after ART, although the causes remains unknown. Obviously the genetic problem plays a considerable role in these debates (6, 7). Some researchers have questioned the genetic impli- cations for offspring of couples having ART and suggested higher incidences of fetal sex-chromosomal aberrations (8) and de novo chromo- somal anomalies (9) after ICSI procedures.

The advent of IVF technique provided a unique opportunity to analyze human preimplantation embryo (10), moreover, cytogenetic analysis of product of conception can be helpful to determine the cause of the pregnancy loss and brings valuable information in the setting of infertility and assisted reproduction (7). However, advanced maternal age, altered karyotype, multiple assisted reproductive technologies failure, repeated miscarriages and spermatozoa obtained by Simon et al. (11) and Magli et al. (12) are characteristics that expose the couples to an increased risk of generat- ing chromosomally abnormal embryos (6).

The main purpose of this study was to evaluate the risks of chromosomal aberrations in ART offsprings of normal karyotype parents by karyotyping these children using their cord blood. Cord blood is widely usable and easy to access; collection is relatively non-invasive and painless. This means for all newborns conceived through ART procedures, cord blood karyotyping may be performed to ensure their normal chromosomal status. On the other hand, there could be some disadvantages such as maternal blood contamination and missing some genetic conditions. Although there are reports based on peripheral blood samples, no study has been reported on such children using their cord blood.

## Materials and Methods

This is a descriptive study that was conducted in the Department of Genetics of Royan Institute, Iran, during January 2009 to January 2012. We considered preparation of umbilical cord blood to be a safe method and therefore, karyotyping was conducted on these samples.

From 88 participating infertile couple candidates for ART procedures, 109 umbilical cord blood samples (68 cases had a singleton, 19 cases had twins and 1 case had a triplet birth) were obtained and analyzed. Prior to commencing the study, ethical approval was received from the local institutional Ethics Committee. When pregnancy happens, a written informed consent was obtained from each couple who participated in the study. A genetic counselor visited all pregnant patients and a pedigree was recorded for each of them. Briefly, data on pregnancies and deliveries (information about ectopic pregnancies, miscarriages, preterm births, stillbirths, live births, multiple pregnancies and terminations) were obtained. Additional clinical findings including history of infertility and reported use of ARTs, maternal ages, date of delivery and presence of congenital abnormalities in the members of the family were also recorded. 

At the time of delivery, the umbilical cord blood samples were collected during cesarean section in sterile heparinized containers and delivered to genetic laboratory within 2 hours. Because of high risks and emergencies in the field of infertility, all samples were obtained by cesarean section based on the gynecologist preference. General pediatric examinations were performed at birth to identify any apparent anatomical abnormalities of the children. In the cytogenetic lab, a trained technician prepared karyotype slides using the Giemsa (G-banding) technique (500-550 bands per karyotype). It should also be mentioned, a drawback of bloodbased G-banding karyotyping is that it can miss extremely subtle chromosome abnormalities that are at the limit of resolution of light microscopy. In brief, approximately 0.5 ml of heparinized whole blood was placed/poured into a glass or plastic tube and inoculated with 10 ml of PB-MAX medium (Gibco, USA). The culture was then incubated at 37°C for 48 hours and thymidine was added at a final concentration of 0.22 μg/ml (0.92 mM) and further incubated for 16 hours in incubator. The culture was subsequently transferred to a centrifuge tube and at 500 xg for 10 minutes. Afterwards, 5 ml of fresh medium without Phytohemagglutinin (PHA) was added and the culture was incubated for an additional 5 hours. Next, the cells were centrifuged as before and washed in fixative for a second time and incubated for 10 minutes. 0.2 μg/ ml of KaryoMAX™ Colcemid Solution (Gibco, USA) was added to each culture tube then the culture was incubated for an additional 15 minutes. Afterwards, the culture was transferred to a centrifuge tube and spun at 500 xg for 10 minutes, then the supernatant was removed and the cells were re-suspended in 10 ml of hypotonic 0.075 M KCl (Gibco, USA) and incubated at 37°C for 15 minutes and then spun at 500 xg for 10 minutes. Subsequently the supernatant was removed, the cellular sediment was agitated and 5-10 ml of fresh, ice-cold fixative made up of 1 part acetic acid to 3 parts methanol was added drop-by-drop, and left in -20°C for 1 hour then spun at 500 xg for 10 minutes. The cell pellet was re-suspended in a small volume 0.5-1 ml of fresh fixative, dropped onto a clean slide and allowed to air dry. At this stage, the slide could be stained with Orecin or Giemsa. Giemsa band- ing has become the most widely used technique in cytogenetic analysis, and the most common method to obtain this staining is to treat slides with Trypsin-EDTA 10X (Gibco, USA). At least 15 metaphases were analyzed per baby and in the case of mosaicism or abnormal karyotype, 50 metaphases were analyzed. The chromosom- al anomalies were reported in accordance with the current international standard nomenclature (13). From 109 newborns, in nine cases prenatal tests and amniocentesis eliminated the need for cord blood karyotyping.

### Statistical analysis

This is a descriptive study and the reported rate is within the range reported in the literature. Abnormal karyotype rates were compared by Fisher’s exact test between ART children and P<0.05 was considered statistically significant. 

## Results

The age of females ranged from 26 to 42 years (mean age of 34 years). Table 1 summarizes family histories of all participating couples in this study (consanguinity, history of spontaneous abortions, ART failure, Intrauterine fetal death (IUFD) in each couple, cleft lip/club foot and mental retardation (MR) in 1^st^ or 2^nd^ cousins). As shown in Table 1, failed ART and IUFD were the most and least frequent features (40.4 and 1.83% respectively) observed in the patients and their extended family.

**Table 1:** Medical history of couples participating in this study


Medical factors	n*	Percentage**

Spontaneous abortions	8	9.1
Consanguinity	28	31.8
ART failure	44	50
IUFD	2	2.3
Cleft lip/club foot in 1^st^ or 2^nd^ cousins	5	5.7
MR in 1^st^ or 2^nd^ cousins	12	13.6


ART; Assisted reproductive technology, MR; Mental retardation, IUFD; Intra Uterus Fetal Death, *; Some of the patients showed more than one medical factor, and **; Percentage of medical factors among 88 studied couples.

**Table 2:** Descriptive characteristics of couples


			

Infertility factor	Female factor	24	27.2
	Male factor	43	48.9
	Male and female factor	14	16
	Idiopathic	7	7.9
Type of infertility	Primary	84	95.45
	Secondary	4	4.55
ART method	IVF	2	2.27
	ICSI	69	78.41
	IUI	17	19.32


ART; Assisted reproductive technology, IVF; *In vitro* fertilization, ICSI; Intra-cytoplasmic sperm injection, and IUI; Intrauterine in- semination.

Descriptive characteristics of couples are summarized in Table 2.

About half of infertility factors were male-based (48.9%) and almost 8% were idiopathic infertiles. The most common infertility treatment was ICSI (78.41%), while IVF is the least used method (2%). And finally, the ratio of primary and secondary infertility was 84% and 4%, respectively. The most and least common sperm retrieval methods were masturbation (53.4%) and retrograde method (2.3%) respectively (Table 3).

**Table 3:** Type of sperm retrieval and their percentages


Type of sperm retrieval	Masturbation	Coitus	PESA	TESE	R.G

Number	47	27	7	5	2
Percentage	53.4	30.7	7.9	5.7	2.3


PESA; Percutaneous epididymal sperm aspiration, TESE; Testicular sperm extraction, and R.G: Retro grade.

**Table 4:** The percentage of chromosome abnormalities (de novo or inherited) in ART children


ART	Children	Normal karyotype (%)	Abnormal karyotype (%)	Total P value

All	105 (96.3)	4 (3.7)		109
		De novo abnormality (%)	Hereditary abnormality (%)	
		1 (0.9)	3 (2.8)	
46,XX	54 (51.43)	2 (1.85)		56
		De novo abnormality (%)	Hereditary abnormality (%)	0.486
		0	2 (100)	
46,XY	51 (48.57)	2 (1.85)		53
		De novo abnormality (%)	Hereditary abnormality (%)	0.486
		1 (50)	1 (50)	


Chromosome analysis was successfully carried out for 109 ART children. As shown in Table 4, for 109 cord blood samples analyzed, the overall rate of abnormality was 3.7% (four cases), and among which, three cases (2.8%) were inherited (one marker chromosome and two inversions) and one case (0.9%) was a de novo chromosome abnormality (structural aberrations). In particular, the inherited cases were a non-identical twin who both showed inversion of chromosome 3 [(46, XX, inv (3) and 46, XY, inv (3)] and a baby who showed a marker chromosome with unknown source, inherited from the mother. In the case of the de novo abnormality, there was a non-identical twin of which the first one was normal (46, XX) and the second one showed translocation between chromosomes 18 and Y [(46, X, t(Y, 18) (q11.2; p11.3)]. The father had a normal karyotype (46, XY) and the baby’s external genitalia was normal. The observed translocation involved Yq11.2 which includes AZF genes, thought to be essential for normal spermatogenesis, thus investigation after puberty was recommended to the parents. Overall, our finding confirmed that there is no significant difference regarding de novo chromosomal abnormality rate in ART children in comparison with naturally conceived babies. Also, ICSI is shown to be applied more in ART babies with chromosomal abnormality. 

## Discussion

There is a growing belief that ART children are phenotypically and somehow genetically different from naturally conceived children. However, the mechanism(s) leading to these possible changes have not been elucidated and may include parental factors, maternal medications, culture media, as well as egg and embryo manipulation (14). The present study included 109 cord blood samples of pregnancies achieved by IVF, ICSI and IUI and was undertaken to examine whether the rate of chromosomal abnormalities is increased among ART conceived children. In our study, we found 0.9% de novo chromosomal abnormality in ART children. This rate compared to the prevalence of this kind of abnormality among naturally conceived newborns in the general population is within the range of 0.7-1% (15). This demonstrates that ART children do not show a higher cytogenetic risk compared to the natural conceived one or in comparison with data from literature in the normal population (7, 16, 17). There are some studies showing the conflicting conclusions in this area (7, 9, 10, 17). Although the incidence of genetic anomalies are high in countries with the higher rate of consanguineous marriage(18-20), our results do not show any increase in the rate of chromosomal abnormalities in babies conceived by consanguineous couples studied here. Also our results are limited to live-born infants and do not involve stillbirths and aborted fetuses.

Several studies suggest abnormal karyotypes in infertile patients and also meiotic aberrations in their germ cells may be considered as the origin of abnormal karyotypes in ART children. These studies reported that the rate of chromosomal ab- normalities in infertile male population has risen above the population baseline and others found a higher incidence of sex chromosome aneuploidy in sperm of men that underwent ICSI (21-23). Casio supposed that an increased incidence of XY spermatozoa was noted in chromosomally normal infertile males, perhaps, due to testicular mosaicism not detected in peripheral blood (6). In 1989, a European survey showed that IVF, compared with natural conception, does not increase the incidence of abortions due to chromosomal abnormality (24). Some authors postulated that the presence of an unbalanced translocation in some gametes may predispose to pre or post implantation failure of embryo development and as a result, the risk of chromosomal abnormalities of ART treatment may be increased (25-28). Genetic chromosomal abnormalities may arise de novo or derive from a familial anomaly present in one of the parents (29). 

Chromosomal aberrations of ART children have been most extensively studied by the Belgian group (9). The limited available data on ICSI fetal karyotypes in comparison with general neonatal population revealed that there is: i. A slight but significant increase in de novo sex chromosome aberrations and structural autosomal abnormalities and ii. An increased number of inherited (mostly from the infertile father) structural aberrations (30- 33). A survey has provided data on the frequency of chromosomal anomalies in newborns after ICSI (23) and found two de novo chromosomal abnormalities (3.6%). They presumed that the other live born children were normal because they noticed no typical malformations consistent with chromosomal defects and that is a percentage of 1.2% (2 out of 167). This was compatible with the prevalence of de novo chromosome abnormalities after ICSI reported in Bonduelle’s study (9).

According to some other studies reporting increased risk of imprinting disorders (34, 35) and malignancies (36) in ART children, these kind of studies at least sound an alarm about the genetic alterations of ART offspring and these procedures should thus be used cautiously.

## Conclusion

Comparing with some reports, our data showed that children born via ART were not subjected to a higher cytogenetic risk than naturally conceived babies in the general population. However, there is conflicting opinion on this area. Since the number of newborns conceived through ART procedures is growing, reports like this must be considered as a pilot study and prenatal tests must be performed for all pregnancies through ART. In lack of amniocentesis, cord blood karyotyping could be perform immediately after birth to find those aberrations which do not have phenotypic alterations such as sex chromosome aneuploidies. Further investigations by array based techniques and epigenetic tests are undergoing to evaluate possible subtle genetic alterations and different epigenetic modifications in ART conceived children.
